# Large-scale comparative genomics to refine the organization of the global *Salmonella enterica* population structure

**DOI:** 10.1099/mgen.0.000906

**Published:** 2022-12-07

**Authors:** Chao Chun Liu, William W. L. Hsiao

**Affiliations:** ^1^​ Department of Molecular Biology and Biochemistry, Simon Fraser University, Burnaby, British Columbia, Canada; ^2^​ Department of Pathology and Laboratory Medicine, University of British Columbia, Vancouver, British Columbia, Canada; ^3^​ Faculty of Health Sciences, Simon Fraser University, Burnaby, British Columbia, Canada

**Keywords:** Bacterial population structure, Bacterial typing nomenclature, Comparative genomics, Molecular evolution, Phylogenetics, *Salmonella*

## Abstract

The White–Kauffmann–Le Minor (WKL) scheme is the most widely used *Salmonella* typing scheme for reporting the disease prevalence of the enteric pathogen. With the advent of whole-genome sequencing (WGS), *in silico* methods have increasingly replaced traditional serotyping due to reproducibility, speed and coverage. However, despite integrating genomic-based typing by *in silico* serotyping tools such as SISTR, *in silico* serotyping in certain contexts remains ambiguous and insufficiently informative. Specifically, *in silico* serotyping does not attempt to resolve polyphyly. Furthermore, in spite of the widespread acknowledgement of polyphyly from genomic studies, the prevalence of polyphyletic serovars is not well characterized. Here, we applied a genomics approach to acquire the necessary resolution to classify genetically discordant serovars and propose an alternative typing scheme that consistently reflect natural *Salmonella* populations. By accessing the unprecedented volume of bacterial genomic data publicly available in GenomeTrakr and PubMLST databases (>180 000 genomes representing 723 serovars), we characterized the global *Salmonella* population structure and systematically identified putative non-monophyletic serovars. The proportion of putative non-monophyletic serovars was estimated higher than previous reports, reinforcing the inability of antigenic determinants to depict the complexity of *Salmonella* evolutionary history. We explored the extent of genetic diversity masked by serotyping labels and found significant intra-serovar molecular differences across many clinically important serovars. To avoid false discovery due to incorrect *in silico* serotyping calls, we cross-referenced reported serovar labels and concluded a low error rate in *in silico* serotyping. The combined application of clustering statistics and genome-wide association methods demonstrated effective characterization of stable bacterial populations and explained functional differences. The collective methods adopted in our study have practical values in establishing genomic-based typing nomenclatures for an entire microbial species or closely related subpopulations. Ultimately, we foresee an improved typing scheme to be a hybrid that integrates both genomic and antigenic information such that the resolution from WGS is leveraged to improve the precision of subpopulation classification while preserving the common names defined by the WKL scheme.

## Data Summary

The assembly accession numbers of the genomes analysed in this study (*n*=204 952) and the associated metadata (e.g. sampling location, collection date, FTP address for data retrieval) are documented in Table S1 (available with the online version of this article). The GenomeTrakr genomes were retrieved from the National Center for Biological Information (NCBI) GenBank database. The PubMLST genomes were retrieved using the BIGSdb API.

The authors confirm all supporting data, code and protocols have been provided within the article or through supplementary data files.

**Impact Statement**

*
Salmonella enterica* (* S. enterica *) is a major foodborne pathogen responsible for an annual incidence rate of more than 90 million cases of foodborne illnesses worldwide. Today, global *Salmonella* surveillance relies heavily on serotyping for disease tracking, risk assessments and data sharing across jurisdictions. However, despite previous *Salmonella* genomic studies reporting discordance between phylogenomic clades and serovars, the prevalence of polyphyletic serovars remains poorly understood. The collective genomic characterization of *Salmonella* thus far, has also yet led to a standardized genomic-based typing method for *Salmonella* longitudinal surveillance that resolves polyphyly. To better understand and address the limitations of *Salmonella* serotyping, we analysed over 180 000 *S. enterica* genomes representing 723 predicted serovars to subdivide the population into evolutionarily stable clusters and propose a stable organization of the *Salmonella* population structure. Analysing nAWC across cgMLST distance thresholds was demonstrated to be an effective means to subdivide large-scale bacterial genomic datasets into stable and functionally divergent clusters that can assist the development of standardized genomic-based typing nomenclatures. The compiled list of non-monophyletic serovars highlighted the previously underestimated prevalence of serotyping inconsistencies and the complexity of *Salmonella* genealogy. By exploring the possibility of erroneous *in silico* serotyping calls, we demonstrated minor discrepancies between reported and predicted serotyping results that could be associated to *in silico* misprediction, laboratory testing error or human errors. More importantly, we observed the urgent need for controlled vocabulary integration in open data settings to strengthen typing data utility and reinforce analysis reproducibility.

## Introduction

*Salmonella enterica* (* S. enterica *) is a significant public health burden that causes more than 90 million cases of foodborne infections globally every year [1]. Existing surveillance networks rely on globally standardized typing nomenclatures to rapidly share information between laboratories, clinicians and public health authorities for risk assessments and disease tracking. Since its introduction more than 80 years ago, *Salmonella* serotyping has been the predominant method for reporting the prevalence of diseases caused by the enteric pathogen. It is considered a low-resolution classification to subdivide the *Salmonella* population into characteristic groups that differ in ecological and clinical features [2]. To date, the White–Kauffmann–Le Minor (WKL) scheme subdivides *S. enterica* into over 2600 serovars based on variations in the O (somatic) and H (flagellar) antigens [3]. Although *Salmonella* serotyping has been established as a central nomenclature for disease reporting and risk assessments, its inconsistencies in the representation of natural microbial populations due to polyphyletic serovars have challenged its role as the gold standard scheme for longitudinal surveillance [4, 5].

Over the years, advances in DNA sequencing have opened up new avenues for microbial typing. Inference of sequence types based on genetic variations in 6–10 housekeeping genes known as multi-locus sequence typing (MLST) has shown high concordance with *Salmonella* serotyping while achieving greater discriminatory power [5]. Highly clonal serovars such as Enteritidis are widely associated with a specific sequence type, while genetically diverse serovars such as Newport and Typhimurium subdivide into multiple sequence types. MLST is considered to be a more robust scheme for natural population characterization than serotyping, as it overcomes the occasional inability of the serotyping scheme to distinguish between genetically unrelated strains [5–8]. In an attempt to develop a novel *Salmonella* typing scheme that better reflects real and stable subpopulations, Achtman *et al*. [5] described the concept of eBurst groups (eBGs) that represent single-linkage chains of sequence types differing by a single locus [5]. A total of 138 eBGs were defined and these clonal complexes effectively segregated polyphyletic serovars into distinguished groups [5]. Although eBG designations have been validated across >100 000 *Salmonella* genomes and determined to be an evolutionarily stable typing scheme [8], its discriminatory power remains limited, given that the scheme only accounts for genetic variations in seven housekeeping genes.

Continual increases in nucleotide sequencing capacity and throughput accompanied by the development of efficient bioinformatic tools render strain differentiation based on a greater number of genetic markers feasible. Adoption of whole-genome sequencing (WGS) enabled the development of core-genome MLST (cgMLST), which extends upon traditional MLST by including 300–3000 loci present in 95–99 % isolates of a bacterial species in the typing schema [9]. And given the conservative nature of the core-genome composition, it represents a stable genomic segment useful for phylogenetic inference [9]. Due to its discriminatory power superior to that of classical fingerprinting methods such as pulsed-field gel electrophoresis (PFGE), phage typing and multi-locus variable-number tandem repeat analysis (MLVA), cgMLST is more effective at differentiating closely related strains, rendering it a compelling complement for outbreak investigations [10, 11]. The development of cgMLST has also inspired improvements to *in silico* serotyping accuracy. SISTR [12], an *in silico* serotyping tool, utilizes a cgMLST scheme consisting of 330 core genes to classify a strain when the tool is not able to classify the strain based on antigenic determinants alone. However, in certain situations, SISTR will classify unrelated lineages as the same serovar because the programme does not attempt to use cgMLST results to resolve polyphyletic serovars [12].

Given the scale of genetic variations surveyed by cgMLST, it has the necessary resolution to define stable *Salmonella* lineages that are consistent with species geneaology. However, defining cgMLST-based lineages requires the establishment of an optimal cgMLST distance threshold (*T*). Herein, we sought to analyse large volumes of cgMLST profiles to establish evolutionarily stable *Salmonella* lineages and subsequently assess the concordance between genomic populations and *in silico* serovar designations. We employed a statistic called neighbourhood adjusted Wallace coefficient (nAWC) [13] that enabled the estimation of an optimal *T* by evaluating cluster stability dynamics in response to clustering thresholds. Our analysis of cluster stability dynamics additionally led to the discovery of differential functional characteristics of diverged subpopulations of a clinically significant serovar. To maximize the coverage and representation of *Salmonella* diversity globally, we compared 180 098 *Salmonella* genomes retrieved from two public databases, namely GenomeTrakr [14] and PubMLST [15]. Reported serovar labels in the metadata were systematically compared against predicted results to estimate the potential impact of erroneous *in silico* serotyping on the analyses conducted in this study, The *S. enterica* population structure revealed by our study can guide surveillance networks to formulate genomic-based *Salmonella* typing nomenclatures [5, 13] that consistently define natural bacterial populations.

## Methods

### Study dataset and quality control

The metadata of the GenomeTrakr *Salmonella* genomes (*n*=186 312) was retrieved on 10 December 2019 using the Pathogen Detection portal (https://www.ncbi.nlm.nih.gov/pathogens), a centralized repository that archives the sample collection information for GenomeTrakr data. The metadata of the PubMLST *Salmonella* genomes (*N*=18 640) was retrieved from the PubMLST database (https://pubmlst.org/) on 9 November 2019. On 13 December 2019, the GenomeTrakr genomes were retrieved from the NCBI GenBank database and the PubMLST genomes were retrieved using the BIGSdb API [15]. The associated metadata of all genomes (*N*=204 952) analysed in this study is made available in Table S1.

The samples were collected from 137 countries (Fig. S1), with the sampling years ranging from 1900 to 2019. As the majority of contributors to the GenomeTrakr WGS network are situated in the USA and the UK, 75 % of the samples were collected from those countries. The samples were sequenced from 4632 different isolation sources distributed across human, environment, animal and food origins, with ~60 % of the samples reported as human clinical samples. A list of all unique isolation sources of the study dataset is provided in Table S2.

quast v5.0.2 [16] and CheckM v1.1.2 [17] were used to conduct data quality assurance by quantifying assembly statistics such as genome size, N50, completeness and contamination. In total, 180 098 genomes fulfilled our data quality criteria and served as the main dataset for all downstream analyses. Assembly quality metrics have been included in Table S1 such that our filtering scheme can be reproduced. Our minimum data quality criteria are as follows: genome length >4 Mbps and <6 Mbps, genome completeness >80 %, genome contamination <5 %, N50 >50 Kbps, contigs count <500, cgMLST profile completeness >95 %.

Mash v2.0 [18] was used to detect sequence duplication and 5906 sequences of the qualified genomes were identified to have zero genetic distance to at least one other sequence in the dataset. These duplicates were not removed from downstream analyses, as they contributed to <5 % of the quality filtered dataset.

### *In silico* serotyping

*Salmonella* serovars were predicted using sistr v1.1.1 [12]. sistr reports different confidence levels of its predictions based on the presence of the antigenic determinants and core genes in the query genome. High confidence *in silico* serovar calls are reported as ‘PASS’ by sistr, and it is determined by the co-presence of the O/H antigenic determinants and >297/330 core genes in the query genome.

### cgMLST and phylogenetic analysis

The cgMLST profile of each genome was generated using chewBACCA v2.0.16 [19] based on a 3246 loci scheme. The original cgMLST scheme was downloaded from https://zenodo.org/record/1323684 and consisted of 3255 loci. Overall, 18 loci appeared to be paralogues due to two or more instances of query coding sequences with multiple secondary alignments reported by ChewBACCA (data not shown). Paralogous loci in the MLST scheme are often assigned non-determined alleles leading to a high degree of missing information in the cgMLST profiles. To minimize missing data, nine paralogous loci that were assigned >100 000 non-determined alleles across the entire dataset, were removed in the final scheme. The remaining paralogous loci on average have <20 000 non-determined alleles assigned across the dataset. Hamming distances were calculated to compare the allelic profile differences and generate a pairwise distance matrix. A neighbour-joining tree was constructed from the pairwise distance matrix using Rapidnj v2.3.2 [20] and visualized in GrapeTree v2.1 [21]. The leaves of the neighbour-joining tree visualized in GrapeTree was collapsed at *T*=25 to consolidate highly similar strains into larger nodes. It was noted that the clustering algorithm of GrapeTree did not yield single-linkage clusters and hence we employed a different clustering algorithm for population structure analysis, explained in the next section. Core-genome SNP phylogenomic analysis of select putative polyphyletic serovars, Choleraesuis and Paratyphi C, was conducted using PhaME v1.0.3 [22] that ran Mummer v3.23 [23] to generate core-genome single nucleotide variant alignments from genome assemblies, and FastTree v2.1.9 [24] to construct a maximum-likelihood tree. The maximum-likelihood tree of Cholerasuis and Paratyphi C genomes was visualized in R using the ggtree v3.2.1 package [25].

### Cluster stability quantification

The adjusted Wallace coefficient was initially proposed to assess cluster concordance and has since been applied to compare the results of different subtyping methods such as PFGE and MLVA [26]. nAWC is an extension to the adjusted Wallace coefficient by assessing cluster concordance between neighbouring cgMLST *T*’s (units in allelic difference) that differ by one distance unit (*T* vs. *T*+1). nAWC ranges from 0 to 1, with the maximum value indicating identical cluster membership between the cluster sets predicted by two neighbouring thresholds [13]. Signatures of cluster stability would arise from the maintenance of high cluster concordance (nAWC >0.99) across successive thresholds. The median values of wide ranges of *T*’s that maintain high cluster concordance were considered suitable for defining evolutionarily stable subpopulations.

TreeCluster [27] was used to cluster the leaves of neighbour-joining trees to generate single-linkage clusters at thresholds ranging from 1 to 3246 allelic differences. The resultant cluster assignments were compared between neighbouring thresholds to calculate nAWC and Shannon entropy using the R scripts available at https://github.com/theInnuendoProject/nAWC.

### Putative non-monophyletic serovar prediction

In addition to cluster membership concordance comparisons, the clusters predicted by TreeCluster were analysed to estimate what proportion of *in silico* serovars are polyphyletic or paraphyletic, collectively referred to as non-monophyletic serovars, henceforth. In a phylogenetic framework, monophyletic groups are characterized by a common evolutionary origin. Hence, monophyletic group members are more genetically related to members of the same taxonomic unit, whereas non-monophyletic groups would violate this notion. The classification of non-monophyletic serovars can be formalized by testing whether inter-serovar cgMLST distance exceeded intra-serovar distance. Specifically, at any given *T,* if genomes of an *in silico* serovar are subdivided into multiple clusters and one of the clusters is a multi-serovar cluster, then we concluded that inter-serovar distance has exceeded intra-serovar distance. By comparing inter- and intra-serovar distances across a wide range of *T*’s instead of a single arbitrary *T*, the method effectively traverses the evolutionary branching of bacterial populations to resolve monophyly. It should also be noted that 201 *in silico* serovars could not be evaluated for monophyly, as these serovars are singletons (one representative genome in the entire dataset); thus, intra-serovar comparisons were not feasible.

### Genome-wide association and functional enrichment

**Fig. 1.**
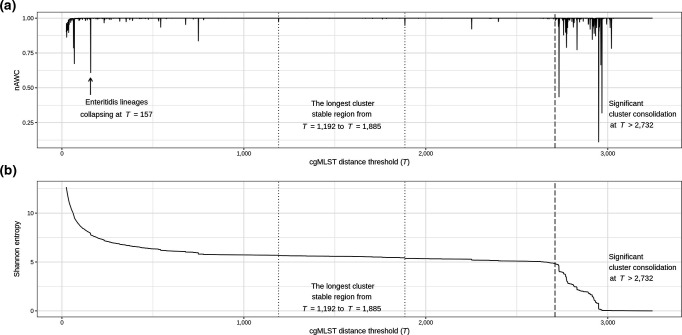
nAWC (a) and Shannon entropy (b) plots calculated across the range of all possible cgMLST distance thresholds.

To explore the functional differences amongst divergent subpopulations revealed by nAWC, we conducted principal component analysis (PCA) and Fisher’s exact test on the cgMLST profiles of two Enteritidis subpopulations responsible for the sharp decrease of nAWC at *T*=157 as labelled in Fig. 1a. The analysis compared 3612 Enteritidis genomes described in Table S3. PCA was conducted in R using the adegenet v2.1.3 package [28]. The principal component loadings were analysed to quantify the allelic contribution to the observed lineage divergence. Fisher’s exact tests were conducted using Scoary v1.6.16 [29] to assess the statistical association between the presence or absence of genotypic features to phenotypic characteristics. The cgMLST profiles were transformed into an allele frequency matrix such that each unique allele of a locus is considered an independent genotypic feature. The Scoary output was filtered by Benjamini–Hochberg corrected *P*-values to determine the set of alleles significantly associated with lineage identity. For pan-genome wide association, Roary v3.12.0 [30] was used to generate a pan-genome gene presence/absence matrix and subsequently used as input to Scoary to identify the statistical association between gene presence and lineage identity. The d*N*/d*S* ratio was calculated using the codeml programme part of the paml v4.9 package [31]. Protein functional annotation enrichment was performed by uploading a list of significant genes to the DAVID web service [32]. Clusters of Orthologous Group (COG) enrichment analysis was performed using the bog v2.0 package [33] in R.

## Results

### Genomic-based characterization of *S. enterica* subpopulations

Quantification of cluster stability by nAWC across the range of possible cgMLST *T* values revealed two notable features: plateaus and peaks (Fig. 1a). The peaks represent significant changes in cluster memberships between neighbouring thresholds due to the consolidation of large clusters. The consolidation of major clusters could be explained by historical event-driven population divergence such as migrations or other bottlenecks. In contrast, the plateaus represent large regions where *T* has negligible effects on cluster memberships. Here, we define a given cluster stable region as any range of thresholds that maintains an nAWC >0.99 for a minimum of five successive *T*’s (*T*, *T*+1, *T*+2, *T*+3, *T*+4). By this definition, we identified a total of 43 cluster stable regions of which the earliest signature of stability occurred at a median value of *T*=45 and the longest region of stability occurred at a median value of *T*=1538. The cluster membership dynamics observed with nAWC closely mirrored the changes in Shannon entropy, a commonly used statistic to quantify population diversity. The consolidations of large clusters observed at *T*>2732 demonstrated by frequent oscillations in nAWC coincided with the sharp decline of Shannon entropy (Fig. 1a, b). The identification of these cluster stable *T*’s provided the means to use genomic data to define the groupings of closely related *Salmonella* isolates.

**Fig. 2.**
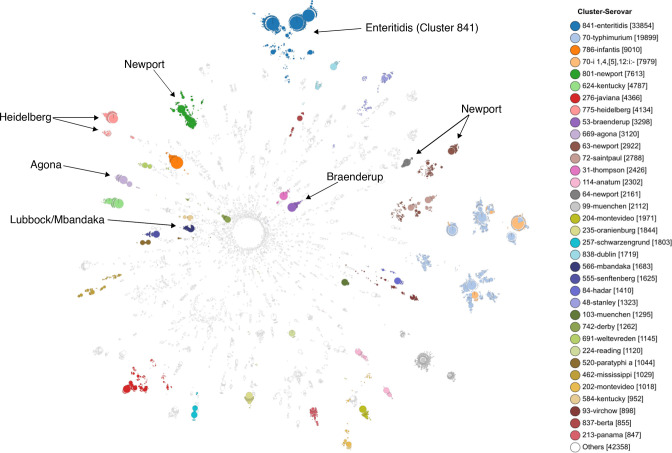
Neighbour-joining tree constructed from 3246-loci cgMLST profiles of 180 098 *Salmonella enterica* genomes. Short distance tree tips (<=25 cgMLST distance) are collapsed to form a larger node with the node size scaled according to the total number of collapsed tips. The nodes are coloured according to serovar and cluster ID assigned at the most stable *T*. Serovars referenced in the main text are highlighted with arrows pointing to their respective nodes. The tree was visualized using GrapeTree [19]. The branch lengths in the visualization are not to scale of the actual lengths.

According to *in silico* serotyping, the 180 098 *Salmonella* genomes represented 723 serovars. To compare between serotyping and genomic-based typing, we first determined the optimal *T to* subdivide the dataset into the most stable genomic clusters, which would occur at the longest cluster stable region from *T=*1192 to *T=*1885. A total of 1342 clusters were predicted at the median *T* of the longest region of stability (*T*=1538). A high degree of concordance was observed between the predicted genomic clusters and *in silico* serovar assignments in which 507/723 (70.1 %) serovars mapped to one specific genomic cluster. This is illustrated by the same cluster code shared across the genomes of the same serovar in Fig. 2. Examples of serovars demonstrating 1-to-1 mapping to genomic clusters included Heidelberg, Braenderup and Agona. In contrast, an evident example of a serovar that did not conform to 1-to-1 mapping is Newport; it is subdivided into numerous genomic clusters: ‘63-Newport’, ‘64-Newport’, ‘801-Newport’ (Fig. 2). Interestingly, 15 of the 20 most prevalent serovars that cause human salmonellosis reported by the United States Centers of Disease Control and Prevention [34] did not exhibit assignments to one specific genomic cluster at the most stable *T*, reflecting the multi-lineage nature of clinically significant serovars, including Enteritidis, Newport, Typhimurium, i 1,4[5],12:i:-, Javiana, Infantis, Muenchen, Montevideo, Thompson, Saintpaul, Oranienburg, Mississippi, Bareilly, Paratyphi B *var*. Java, and Anatum.

In contrast to previous reports, which described the serovar Enteritidis to be sequence-type-specific and highly clonal [35], we found the Enteritidis isolates to segregate into eight different clusters at the most stable *T*. However, significant bias was indeed observed in the relative abundance across the Enteritidis clusters. The global Enteritidis strains appeared to have expanded from an epidemic clone, as 33 980 (99.4 %) Enteritidis isolates in the dataset were classified under a single cluster, Cluster 841. It is unlikely that the minor clusters were mispredicted due to data quality, as their average genome completeness and contamination were within 1 % difference from that of Cluster 841. Hence, these minor clusters likely represent real Enteritidis subpopulations.

To distinguish between uniform and skewed cluster distribution across these multi-lineage serovars, we compared the Shannon entropy of each clinically significant serovar at the most stable *T* (Fig. S2). Of the clinically significant serovars, Bareilly, Javiana, Mississippi, Montevideo, Muenchen, Newport, Oranienburg, Paratyphi B *var*. Java, Saintpaul and Thompson more closely approximated uniform cluster distribution (Shannon entropy **≈** 1), indicating that the lineages of these serovars are equally prevalent unlike Enteritidis.

Given the clustering data at the most stable *T*, we systematically identified serovars of a common lineage by searching for instances of multi-serovars clustering. Overall, 452 of the 1342 (33.7 %) genomic clusters were found to consist of two or more different *in silico* serovars, suggesting that each grouping of these ‘sister-serovars’ share stable genomic contents. Examples included the shared lineage between Lubbock and Mbandaka, the inter-serogroup clustering of the serovars Bredeney (B), Give (E1), Kimuenza (B) and Schwarzengrund (B), as well as the within-serogroup B clustering of the serovars Brandenburg, Reading, Madras and Sandiego. We have provided the cluster assignments defined at the median *T* of the 43 different cluster stable regions in Table S1 to enable queries of genetically related serovars.

### Characterization of genomically similar serovars

Although existing *in silico* serotyping tools leverage the correlation between genome-wide variations and serovars for *in silico* prediction, this observed correlation has yet been systematically evaluated across most serovars on a large scale. We began by characterizing a list of genomically similar serovars to assess whether genomically similar serovars also share similar antigenic combinations. We defined pairs of genomically similar serovars based on the clustering observed at the earliest signature of stability (*T*=45). In this region, a total of 23 748 clusters were predicted, of which 23 584 (99.31 %) clusters contained isolates of a single *in silico* serovar. Subsequent investigation of the other 164 (0.69 %) clusters led to the identification of a total of 65 genomically similar serovar pairs (Fig. S3). Low confidence *in silico* serovar predictions were excluded when determining compositionally mixed clusters to avoid inferring artificial relationships due to erroneous *in silico* serotyping. As expected, phenotypic phase variants of Typhimurium and Paratyphi B/Paratyphi B variant Java were amongst the identified serovar pairs that shared highly similar core genomes (Fig. S3). We observed a total of 19 genomically similar serovar pairs with different O-antigens (i.e. different serogroups) and four serovar pairs that differed by two or more antigenic types (Fig. S3). The serovar pairs that shared dissimilar antigens primarily comprised of rarely sampled serovars such as Madras, Winterthur and Moscow. Due to the sparse sampling of these serovars, there was insufficient evidence to suggest a dissociation between antigenic and genomic variation.

In contrast to another study [36], our analysis did not identify several genetically similar serovar pairs previously reported to share identical PFGE patterns. We discovered that the insensitivity was due to consistent discrepancies between sistr serovar predictions and the reported serovars in the metadata (Table S4). Notably, sistr could not distinguish between the O-6 antigen serovar pairs reported by Mikoleit *et al*. [36], which led to our inability to detect select genetically similar O-6 antigen serovar pairs.

### Characterization of putative non-monophyletic serovars

We next systematically characterized putative non-monophyletic serovars that represent genetically inconsistent classifications. Numerous large-scale genomic studies have recently investigated *S. enterica* population structure and reported a list of polyphyletic serovars [37, 38]. However, we argue that these studies underestimated the prevalence of polyphyletic serovars, as the studies either focused solely on clinically significant serovars or analysed a select number of genomes for each serovar; thus, the studies did not account for the true range of genetic diversity.

Using the approach described in Methods - Putative non-monophyletic serovar prediction, we found 265/537 (49.3 %) putative non-monophyletic serovars, including Enteritidis, Typhimurium, Newport, Kentucky and Javiana. Subsequent manual literature curation indicated that 18 % of the evaluated serovars had previously been reported to be polyphyletic or paraphyletic [5, 37–48]. To confirm the validity of our approach to predict monophyly, we performed maximum-likelihood-based phylogenetic reconstruction of two serovars Choleraesuis and Paratyphi C that were predicted as non-monophyletic by our approach, but previously reported as monophyletic [49]. The resulting phylogeny demonstrated two major branches that segregated strains of the two serovars (Fig. S4). However, several sparsely sampled lineages of the two serovars were observed not to share a recent common ancestor of the two major branches, suggesting Cholaerasuis and Paratyphi C are polyphyletic serovars.

**Fig. 3.**
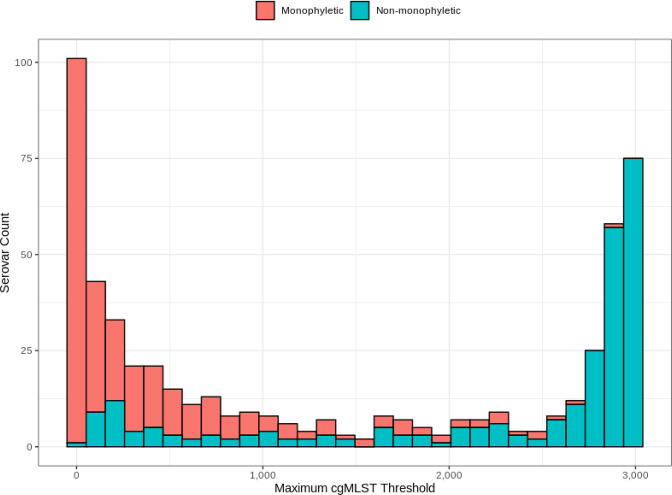
Distribution of monophyletic and non-monophyletic serovars by the maximum cgMLST allelic distance required to merge all genomes of the same serovar in a single-linkage cluster.

To investigate the extent of genetic variation within a given serovar and the consistency of genetic variability across all serovars, we evaluated the maximum cgMLST allelic distance required to link genomes of the same *in silico* serovar assignments in a single cluster. We found genomes of 209/537 (38.9 %) serovars required a distance of >2000 allelic differences to be linked in a single cluster (Fig. 3). Moreover, serovars with greater maximum linkage distance appeared more likely to be predicted as non-monophyletic (Fig. 3). The higher than previously reported prevalence of putative non-monophyletic serovars and the multitude of multi-lineage serovars demonstrated from our collective analyses express the complex nature of *Salmonella* evolutionary history, which is not reflected by antigenic determinants. Lastly, we have found evidence to suggest that our analysis has underestimated the true proportion of putative non-monophyletic serovars, as the serovar sample size was found to be a statistically significant predictor of non-monophyly (Fig. S5).

### Analysis of cluster destabilizing thresholds

**Fig. 4.**
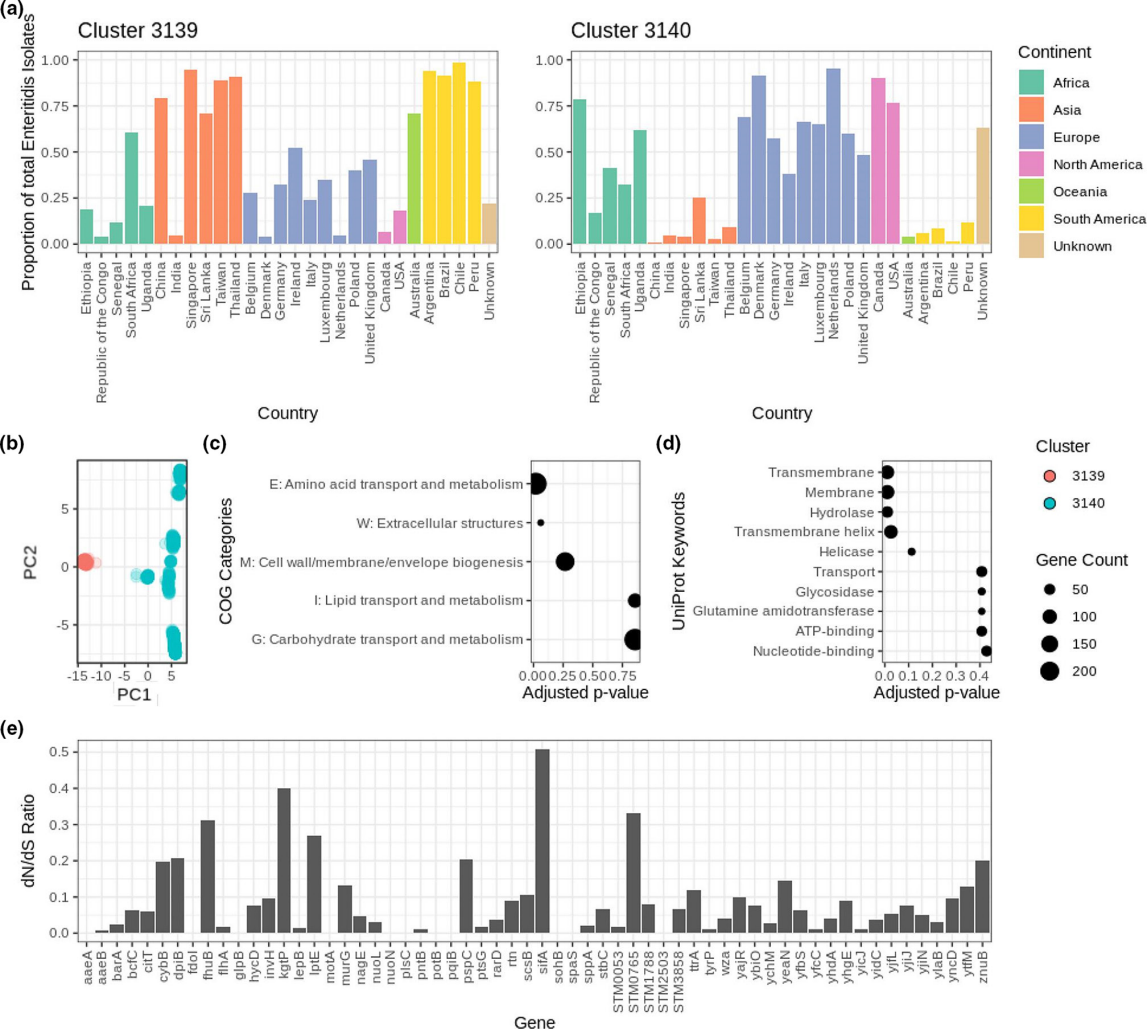
Genomic analysis of geographically segregated Enteritidis subpopulations. (a) Geographical distribution of Enteritidis isolates classified in Clusters 3139 and 3140 with the proportion calculated by dividing the number of isolates from each country classified as Cluster 3139 or 3140 by the total number of Enteritidis isolates from the same country in the entire dataset. (b) PCA analysis of the cgMLST profiles of isolates from the two Enteritidis subpopulations. (c, d) Functional annotation enrichment in the top COG categories and UniProt keywords ranked by adjusted *P*-values. (e) d*N*/d*S* ratio of the membrane protein genes found to exhibit lineage-specific variation across the two Enteritidis subpopulations.

Thus far, we have explored the usage of nAWC to identify *T*’s to subdivide *Salmonella* genomic data into stable clusters. The other significant feature revealed from the nAWC curve (Fig. 1a) was the peaks that signified the consolidation of divergent lineages, referred to as ‘cluster destabilizing thresholds’, henceforth. We investigated the clustering dynamics at one of these cluster destabilizing thresholds to explore the possibility of discovering additional subpopulation structures that could expand our understanding of *Salmonella* evolutionary history. The cluster destabilizing threshold at *T*=157 (Fig. 1a) was arbitrarily chosen for analysis. It was determined that the decrease in nAWC was due to the convergence of two major Enteritidis clusters named ‘Cluster 3139’ (*N*=10 546) and ‘Cluster 3140’ (*N*=21 398). This was confirmed by recomputing nAWC at *T=*157 while excluding the Enteritidis isolates from the predicted cluster, which yielded an nAWC value above 0.99. Comparing the geographic distribution of the two Enteritidis clusters revealed geographical segregation of the two clusters (Fig. 4a). Log ratio (LR) was calculated to compare the relative frequencies in which an isolate from the two clusters was sampled from a particular continent. Cluster 3139 isolates predominated in Asia (LR: 3.13), South America (LR: 3.87) and Oceania (LR: 4.16), while Cluster 3140 isolates predominated in North America (LR: 2.08). As an orthogonal validation, a total of 3612 genomes were randomly selected from the two clusters for PCA, which similarly revealed the genetic divergence of the two subpopulations that can be explained by the first principal component alone (Fig. 4b). The inclusion of the second principal component revealed the partition of Cluster 3140 isolates into additional subpopulations.

To rationalize the factors that contribute to the genetic fixation in the subpopulations, genome-wide association analysis was conducted to identify the genetic variations that distinguish the two Enteritidis clusters. The allelic profiles of the Enteritidis isolates were transformed into an allele frequency matrix (3612×25 771), and the lineage-specific alleles were inferred using statistical tests and PCA, as described in Methods - Genome-wide association and functional enrichment. Analysis of the principal component loadings identified 284 different alleles involving 168 loci that showed significant contributions (loading threshold >0.025) to the observed genetic segregation of the two Enteritidis subpopulations. An independent analysis of the core-genome allele association to cluster identity using Scoary [29] suggested a higher number of alleles in which 388 alleles involving 183 loci demonstrated significant statistical association (adjusted *P*-value<0.05) to Enteritidis subpopulation classification. High concordance was observed between the two methods, as all 168 loci predicted by PCA were also identified in the Scoary analysis. Amongst the significant loci, the cpsG gene matched the minimal SNP-based subtyping scheme previously proposed to assist the differentiation of the two major Enteritidis lineages based on two genes, cpsG and citT [49]. The significant genes were found functionally enriched in COG category E (amino acid transport and metabolism) and membrane protein genes (Fig. 4c, d). Targeted assessment of the non-synonymous (d*N*) and synonymous (d*S*) mutation rates of the membrane protein genes revealed a d*N*/d*S* ratio <1 across all membrane protein genes (Fig. 4e). Pan-genome-wide association analysis led to the discovery of six genes uniquely carried by Cluster 3139 isolates with functions in translation, membrane transport and DNA recombination (Table S5). Additionally, six other genes were found to be uniquely carried by Cluster 3140 isolates with functions in DNA recombination, metabolism and prophage assembly (Table S5).

### Comparison of sistr *in silico* serotyping to reported serovars

Our estimation of inter- and intra-serovar similarity could be significantly confounded by erroneous *in silico* serotyping resulting in the misclassification of non-monophyly. Hence, we took the opportunity to evaluate 102 157 public *Salmonella* genomes archived with serovar information to assess the consistency between sistr *in silico* serotyping and reported serotypes found in public databases. Based on strict string matching, an overall 90.6 % concordance between predicted serovars and reported serovars was observed. We noticed a higher rate of discrepancy in lower-quality genome assemblies (< 297/330 core genes found by sistr), in which discrepancies were detected in 22 % of lower-quality genomes compared to 9.2 % in high-quality genomes. Further analysis of the discrepancies in high-quality genomes revealed sources of inconsistencies unrelated to data quality. Altogether, 70.4 % of the observed serovar discrepancies in high-quality genomes occurred due to the lack of data harmonization for serovar information in public sequence databases. The inconsistent use of different phrases to describe the same serovar and spelling errors were frequently observed. A prominent example is the monophasic variant of Typhimurium, which was reported in more than eight different ways in the metadata including “I 1,4,[5],12:i:-”, ‘typhimurium monophasic’, ‘typhimurium - monophasic’, “4,[5],12:i:-”, “1,4,[5],12:i:-”, “I 4,5,12:i:-”, “4,5,12:i”, and “4,5,12:i-”. However, the remaining 29.6 % of the discrepancies in high-quality genomes are likely unrelated to data quality or semantics, and instead related to potential errors in *in silico* serovar prediction or reports in public databases. Examples include the aforementioned O-6 antigen serovar pairs and 1086 other unique mismatches listed in Table S6. Following the correction for discrepancies due to data quality and semantics, the overall concordance rate between serotyping reports and predictions increased to 96.9 %, suggesting that misclassification by *in silico* serotyping has a relatively minor effect on our analyses.

## Discussion

From the cgMLST analysis of a diverse *Salmonella* dataset of 180 098 genomes, we demonstrated that the global population structure of the enteric pathogen can be arranged into 1342 stable subpopulations. The robustness of our cluster definition is derived from the fact that the cluster assignments, richness and evenness remain stable across a wide range of distance cutoffs. Moreover, the validity of our population structure analysis can be demonstrated from the evident correlation between serotyping and genomic variation observed from the phylogenetic tree (Fig. 2). Our large-scale phylogenetic analysis also echoed numerous observations reported in the literature. Examples included the identification of serovars of a common descent: Lubbock and Mbandaka [50], the geographical segregation between the Enteritidis lineages previously described by Deng *et al*. [51], and the greater diversification observed in the North American Enteritidis lineage (Cluster 3140) [49].

The observation that many serovars were subdivided into multiple lineages was consistent with our expectation, considering that the WKL scheme was intended to serve as a low-resolution typing method [5]. However, the inconsistencies of *Salmonella* serotyping became apparent when the extent of genetic variability in each serovar was evaluated. Our results suggested that there are many genetically inconsistent serovars that do not represent groupings of genetically related isolates. Thus, their corresponding isolates likely do not share the same epidemiological or clinical implications. Our findings agreed with a previous report that described the failure of the serovar Senftenberg to represent a classification of genetically related isolates [52]. Specifically, different subsets of the serovar Senftenberg isolates were reported to share either the same sequence type (ST14) with the serovar Westhampton or the same sequence type (ST185) with another serovar, Dessau [52]. For future work, we suggest a systematic analysis of serovar inconsistencies in a phylogenetic context to confirm the non-monophyletic nature of the serovars that we have identified solely based on genetic distances.

Although a correlation indeed exists between antigenic and genomic variations, caution must be made surrounding specific serovars to avoid misinterpretations of biological, ecological and epidemiological features based on serotyping. It can be postulated that the occasional discrepancy between antigenic and genome-wide variation is due to the homologous recombination of genes involved in antigen synthesis, which can confound the inference of genealogy. Recently, it has been reported that the O-antigen biosynthesis genes are situated in a genomic island with distinctive GC content rendering it a hot spot for homologous recombination and horizontal gene transfer [53].

Our discovery of over 200 serovars that had not been reported as putative non-monophyletic classifications is a further indication of the negligence of genomic investigations for many serovars thus far. Moreover, previous studies often selected a minor subset of genomes to represent a given serovar leading to insufficient coverage of genetic diversity and consequently the assumption of monophyly [37, 38, 48, 54]. Hence, the collective analysis of a highly genetically diverse dataset on the scale of hundreds of thousands of genomes remains valuable to deduce a more holistic view of bacterial population structures. To contribute to the knowledge base of *Salmonella* serotyping, we compiled a public resource (Table S7) that documents the putative non-monophyletic serovars, as well as the serovars that belong to the same lineage as the putative non-monophyletic serovars.

The deficiencies of serotyping for longitudinal surveillance can be addressed by WGS, given its capability to survey genome-wide markers and thereby empower genomics researchers to model the divergence of bacterial populations with greater precision and resolution. By subdividing *Salmonella* into 1342 groups based on the most stable *T*, a genomic-based typing scheme can be developed. A single sequence can be selected from each stable cluster to construct a set of reference sequences that collectively represent the global *Salmonella* population structure. Subsequent alignments of query sequences to the reference sequences would thus inform the subtypes of the query genomes based on the most similar reference.

In fact, a similar solution has already been implemented in Enterobase that integrates the application of cgMLST *T*’s to define stable *Salmonella* clusters represented by hierarchical cluster codes [55]. The Enterobase cgMLST thresholds have been specifically chosen with the intention to reflect natural *Salmonella* subpopulations such as subspecies, superlineages and eBGs [55]. Integrating hierarchical cluster code assignments in Enterobase represents an initial step towards establishing a standardized WGS-based typing scheme for *Salmonella *. An alternative solution is major antigenic cluter typing introduced by Chattaway *et al*. [56] that merges the nomenclatures of serotyping and MLST typing in an attempt to resolve problematic serovars [56]. One primary concern with the adoption of genomic-based typing nomenclature has been surrounding the incomparability between genomic typing results to historic surveillance data. To address this concern, genomic-based typing nomenclatures should preserve the serovar names defined by the WKL scheme and devise a naming system that effectively integrates both genomic and serotyping information.

With a multitude of genomic solutions available, the next steps towards global standardization should include community convergence on a universal genomic-based typing scheme and collaborative efforts to characterize the functional differences, ecological adaptations and health risks associated with each subpopulation in order to maximize the utility of genomic-based typing. Moreover, community consensus on a core- or whole-genome MLST scheme is critical to converge on a fixed set of genome-wide loci for comparison and reinforce a standardized allele number nomenclature. In particular, the Chewie Nomenclature Server (https://chewbbaca.online/) could be a highly promising avenue to synchronize local schema with globally maintained schema and centralize a collection of gene-by-gene subtyping schema for microbial pathogens [57].

The importance of standardization and data harmonization cannot be undermined, as the lack of controlled vocabularies in open data settings hinders data interpretability and summarization. The high degree of metadata inconsistencies in public foodborne pathogen databases is evident from the frequent discrepancies between sistr predictions and reported serovars solely due to semantics. The unrestrictive entry of serovar information in GenomeTrakr and PubMLST hindered the accurate estimation of the number of serovars in the study dataset based on the reported serovars in the metadata alone. Moreover, it rendered the estimated 90.6 % concordance rate between sistr predictions and reported serovars to likely represent an underestimation of the true concordance rate. Our uncorrected concordance rate was in fact, marginally lower than a previous estimate of 91.91 % [58]. The integration of controlled vocabularies for typing information in public databases would be highly valuable for future studies that intend to analyse public *Salmonella* sequences by eliminating the need for time-consuming data harmonization and thus reinforcing analysis reproducibility.

To explore the application of nAWC to reveal cluster consolidation dynamics and inform evolutionary histories, we analysed the genomic differences between two geographically segregated Enteritidis lineages that converged at a cluster destabilizing threshold. The pronounced allele frequency bias across different geographical regions demonstrated from genome-wide association analyses suggested that the observed lineage divergence could be a consequence of niche adaptation or founder effects. A previous study on the evolutionary history of *Salmonella* Enteritidis had estimated the most recent common ancestor of present-day Enteritidis lineages to have existed around the 1600s when cross-continental overseas trades were increasingly embraced [51]. Hence, we hypothesize that an ancestral European Enteritidis subpopulation had migrated to America during the era of colonial trade and evolved in reproductive isolation leading to the fixation of the lineage-specific alleles observed in Cluster 3139. However, the enrichment of allelic bias in metabolic and membrane transport functions could reflect the local adaptation of the Enteritidis subpopulations to variable nutrient availability in different regions, a phenomenon that has been previously reported in a metabolic analysis of Enteritidis lineages in high- and low-income settings [59]. We recommend a future study of *in vitro* metabolic activities of the two Enteritidis populations and correlating differential metabolic activities to the divergence of lineage-specific alleles to test our hypotheses.

In the genomics era, the development of efficient bioinformatic tools and high-throughput sequencing has revolutionized our understanding of microbial evolution and biology. Here, we continued to echo the value of genomic data by proposing systematic ways to define the global *Salmonella* population structure and discovering novel genetic patterns that differentiate microbial subpopulations. Our study is a further demonstration of how global genomic data sharing benefits scientific research. The increasing open accessibility of large-scale genomic datasets with minimal metadata fields will foster new directions to investigate molecular evolution and microbial diversity. With the growing integration of genomics in infectious disease surveillance worldwide, we encourage joint efforts across scientific research and public health to standardize a genomic-based typing nomenclature for global disease reporting such that *Salmonella* typing consistently reflect both genetic relatedness and epidemiological significance.

## Supplementary Data

Supplementary material 1

Supplementary material 2
