# Supplementary material for: Large-scale comparative genomics to refine the organization of the global Salmonella enterica population structure
Source: Microb Genom. 2022 Dec 7;8(12):mgen000906. doi: 10.1099/mgen.0.000906 (PMC9837569; doi:10.1099/mgen.0.000906)
Supplement: Supplementary material 1 [file mgen-8-906-s001.pdf]

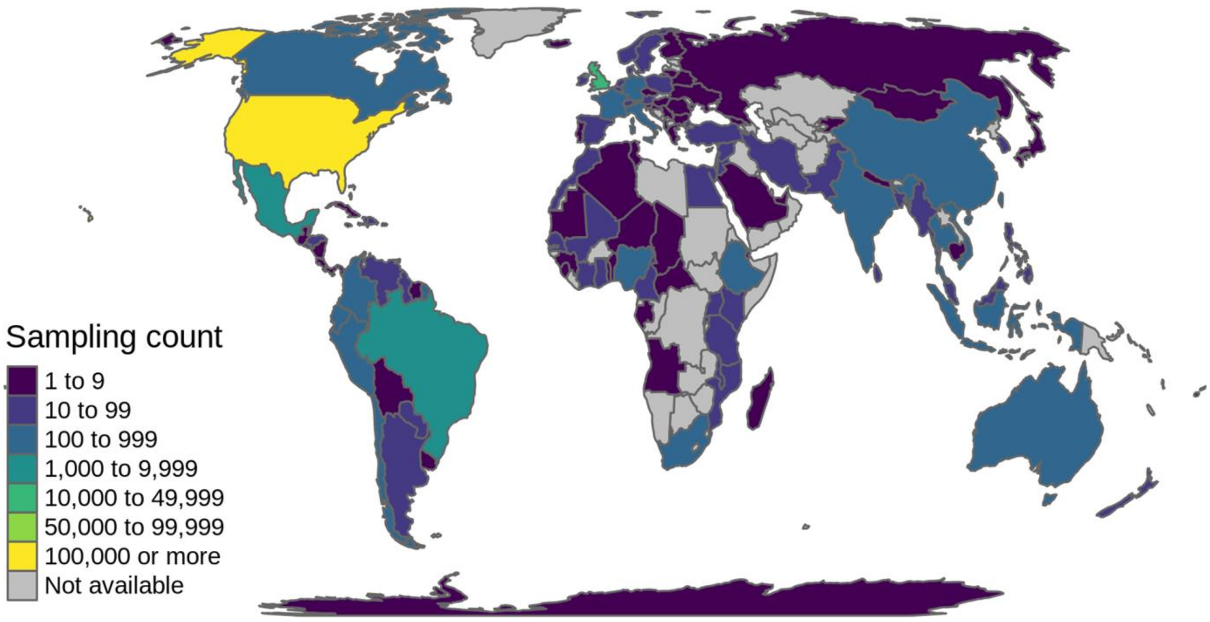


**Fig. S1.** Geographical distribution of the study dataset. The geographical coverage spanned 137 countries from all six continents. As the majority of contributors to the GenomeTrakr WGS network are situated in the United States and the United Kingdom, 75% of the samples were collected from those countries.

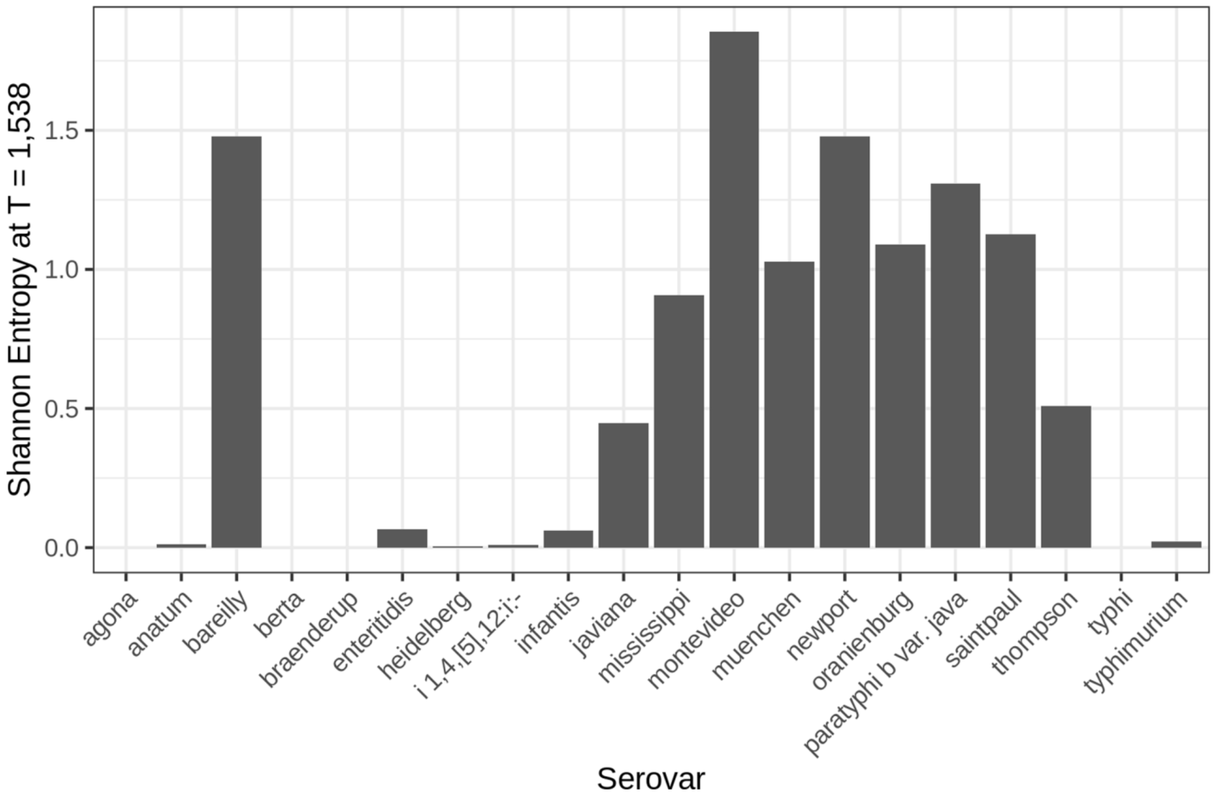

**Fig. S2.** Shannon entropy of the top 20 most prevalent *Salmonella serovars* according to the US CDC [32] calculated based on the genomic clusters predicted at $T$ = 1,538. Bareilly, Javiana, Mississippi, Montevideo, Muenchen, Newport, Oranienburg, Paratyphi B var. Java, Saintpaul, and Thompson were amongst the top 20 serovars that closely approximated uniform cluster distribution (Shannon entropy ≈ 1).

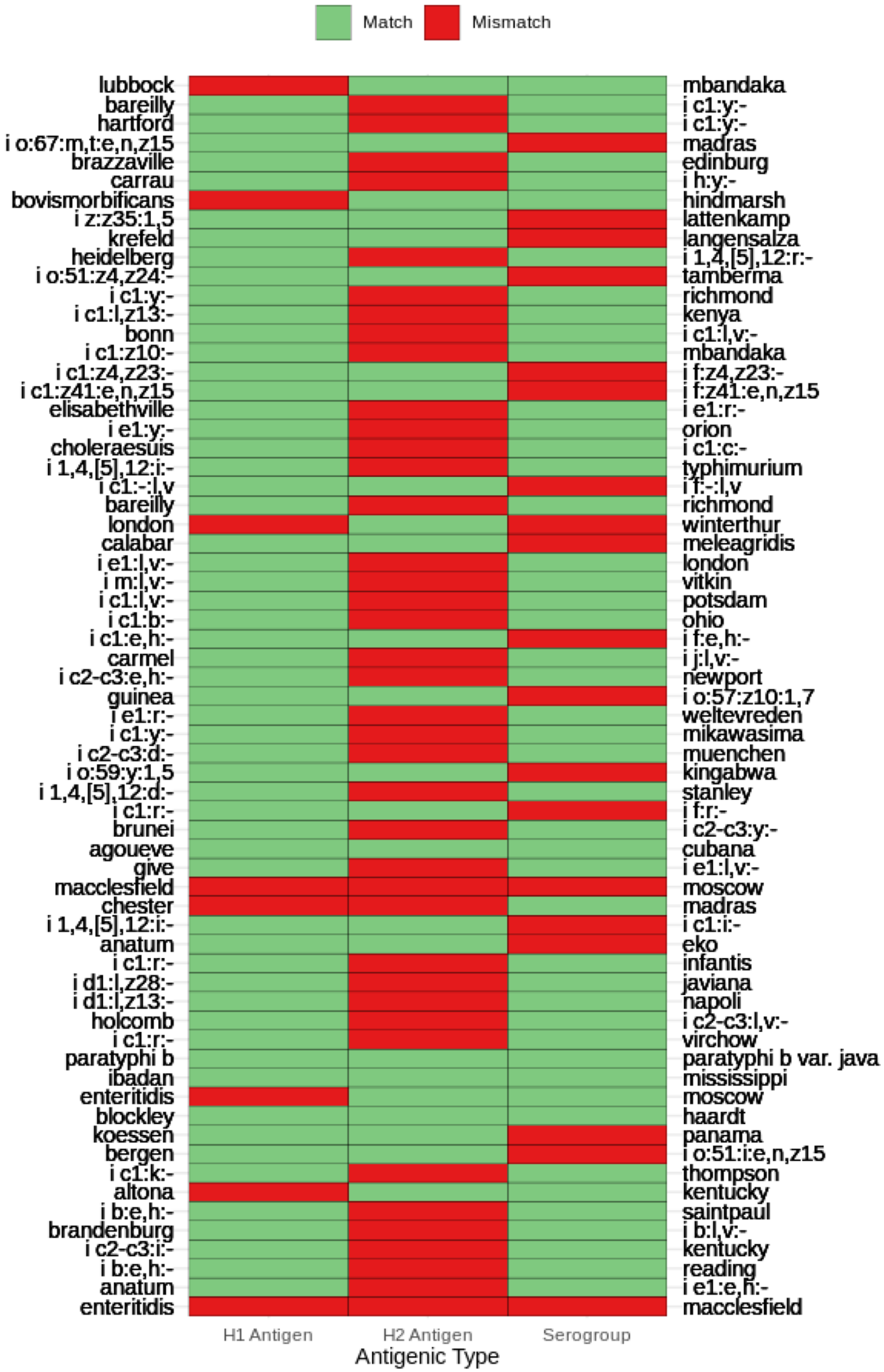
Match
Mismatch
lubbock
bareilly
hartford
i o:67:m,t:e,n,z15
brazzaville
carrau
bovismorbificans
i z:z35:1,5
krefeld
heidelberg
i o:51:z4,z24:-
i c1:y:-
i c1:l,z13:-
bonn
i c1:z10:-
i c1:z4,z23:-
i c1:z41:e,n,z15
elisabethville
i e1:y:-
choleraesuis
i 1,4,[5],12:i:-
i c1:-:l,v
bareilly
london
calabar
i e1:l,v:-
i m:l,v:-
i c1:l,v:-
i c1:b:-
i c1:e,h:-
carmel
i c2-c3:e,h:-
guinea
i e1:r:-
i c1:y:-
i c2-c3:d:-
i o:59:y:1,5
i 1,4,[5],12:d:-
i c1:r:-
brunei
agoueve
give
macclesfield
chester
i 1,4,[5],12:i:-
anatum
i c1:r:-
i d1:l,z28:-
i d1:l,z13:-
holcomb
i c1:r:-
paratyphi b
ibadan
enteritidis
blockley
koessen
bergen
i c1:k:-
altona
i b:e,h:-
brandenburg
i c2-c3:i:-
i b:e,h:-
anatum
enteritidis
mbandaka
i c1:y:-
i c1:y:-
madras
edinburg
i h:y:-
hindmarsh
lattenkamp
langensalza
i 1,4,[5],12:r:-
tamberma
richmond
kenya
i c1:l,v:-
mbandaka
i f:z4,z23:-
i f:z41:e,n,z15
i e1:r:-
orion
i c1:c:-
typhimurium
i f:-:l,v
richmond
winterthur
meleagridis
london
vitkin
potsdam
ohio
i f:e,h:-
i j:l,v:-
newport
i o:57:z10:1,7
weltevreden
mikawasima
muenchen
kingabwa
stanley
i f:r:-
i c2-c3:y:-
cubana
i e1:l,v:-
moscow
madras
i c1:i:-
eko
infantis
javiana
napoli
i c2-c3:l,v:-
virchow
paratyphi b var. java
mississippi
moscow
haardt
panama
i o:51:i:e,n,z15
thompson
kentucky
saintpaul
i b:l,v:-
kentucky
reading
i e1:e,h:-
macclesfield
H1 Antigen
H2 Antigen
Serogroup
Antigenic Type

**Fig. S3.** Antigenic formula comparisons of 65 genomically similar serovar pairs. Four serovar pairs were found to be different in two or more antigen types. However, due to the sparse sampling of the four serovar pairs, there was insufficient evidence to suggest a dissociation between antigenic and genomic variation.

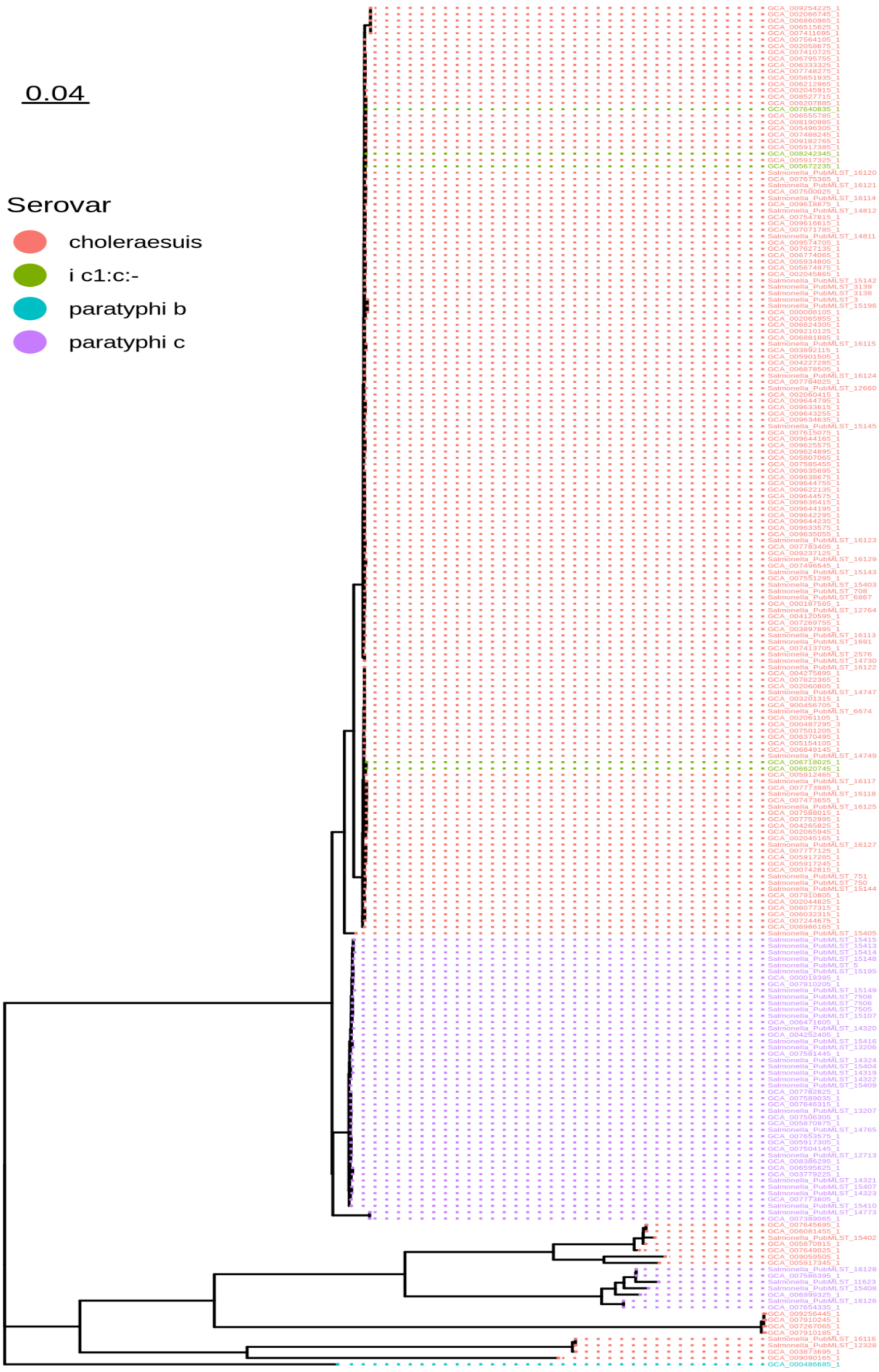


**Fig. S4.** Maximum likelihood tree of serovars Choleraesuis, Paratyphi C, and i c1:c:- genomes rooted by a Paratyphi B outgroup (genome accession: GCA_000486685.1) and visualized in R. The tree revealed two major branches that segregated Choleraesuis and Paratyphi C strains. However, several

sparsely sampled lineages of the two serovars were observed not to share a recent common ancestor of the two major branches. The tree topology suggested that Cholaerasuis and Paratyphi C are polyphyletic serovars in contrast to previous reports [49].

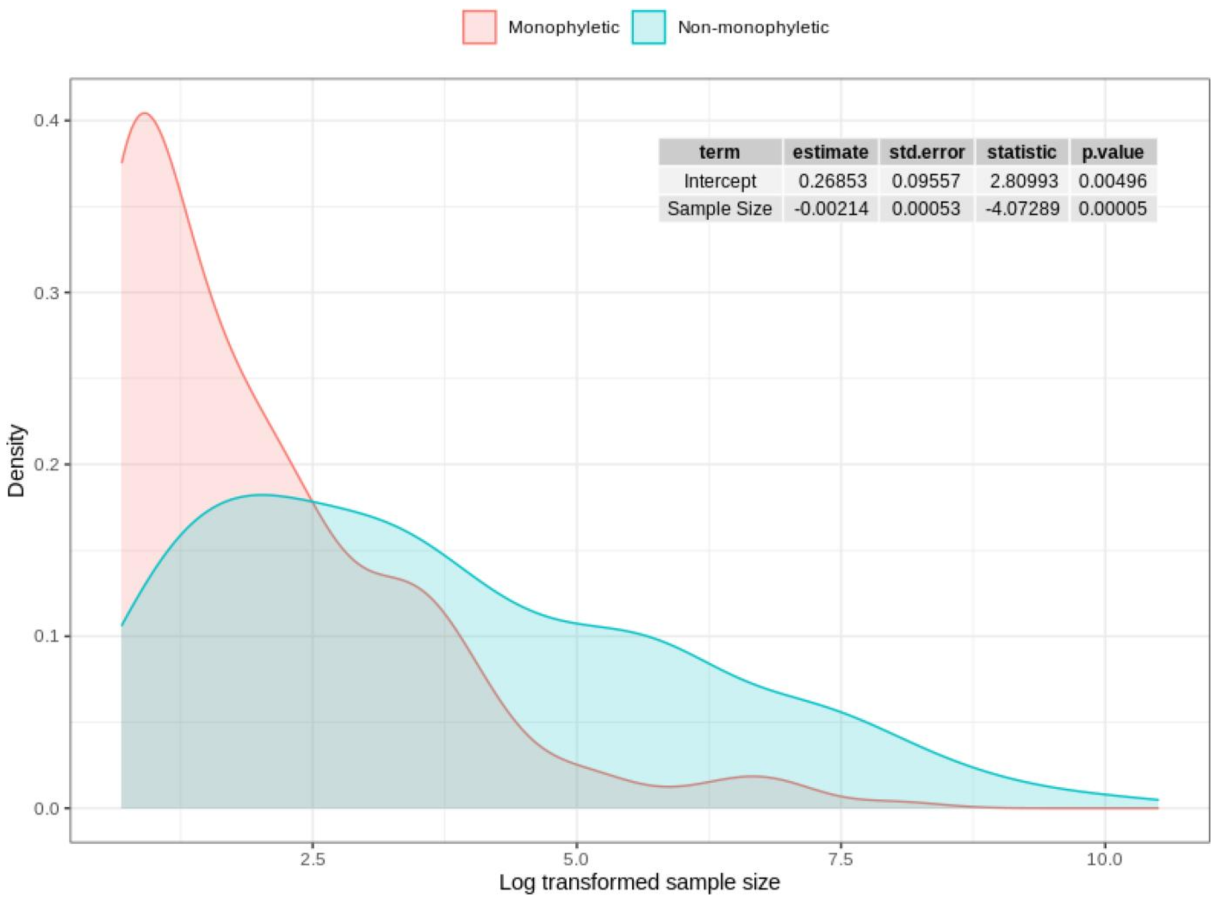


| term | estimate | std.error | statistic | p.value |
|---|---|---|---|---|
| Intercept | 0.26853 | 0.09557 | 2.80993 | 0.00496 |
| Sample Size | -0.00214 | 0.00053 | -4.07289 | 0.00005 |

**Fig. S5.** Log-transformed sample size density distribution of predicted monophyletic and non-monophyletic serovars. The summary table shows the statistical significance and regression coefficient of serovar sample size as a predictor of monophyly estimated by logistic regression. The statistically significant association observed between monophyly and serovar sample size suggested that our estimation of the true proportion of putative non-monophyletic serovars is likely an underestimate.
